# Sex difference in the associations among obesity-related indices with incidence of diabetes mellitus in a large Taiwanese population follow-up study

**DOI:** 10.3389/fpubh.2023.1094471

**Published:** 2023-01-20

**Authors:** Tung-Ling Chung, Yi-Hsueh Liu, Pei-Yu Wu, Jiun-Chi Huang, Szu-Chia Chen

**Affiliations:** ^1^Graduate Institute of Medicine, College of Medicine, Kaohsiung Medical University, Kaohsiung, Taiwan; ^2^Division of Nephrology, Department of Internal Medicine, Kaohsiung Veterans General Hospital, Kaohsiung, Taiwan; ^3^Division of Cardiology, Department of Internal Medicine, Kaohsiung Medical University Hospital, Kaohsiung, Taiwan; ^4^Department of Internal Medicine, Kaohsiung Municipal Siaogang Hospital, Kaohsiung Medical University, Kaohsiung, Taiwan; ^5^Division of Nephrology, Department of Internal Medicine, Kaohsiung Medical University Hospital, Kaohsiung Medical University, Kaohsiung, Taiwan; ^6^Faculty of Medicine, College of Medicine, Kaohsiung Medical University, Kaohsiung, Taiwan; ^7^Research Center for Precision Environmental Medicine, Kaohsiung Medical University, Kaohsiung, Taiwan

**Keywords:** obesity-related index, sex difference, incident diabetes mellitus, Taiwan biobank, follow-up

## Abstract

**Background:**

Obesity is a major risk factor for diabetes mellitus (DM), which is in turn a major risk factor for cardiovascular diseases such as coronary artery disease and stroke. As few studies have investigated sex differences in the association between obesity and incidence of DM, the aim of this longitudinal study was to explore this issue in a large group of Taiwanese participants.

**Methods:**

A total of 24,346 participants were enrolled in this study, of whom 8,334 (mean age, 50.6 ± 11.0 years) were male and 16,012 (mean age, 50.5 ± 10.1 years) were female. The following obesity-related indices were studied: body mass index, waist-to-height ratio, waist-to-hip ratio (WHR), body roundness index, conicity index (CI), body adiposity index, abdominal volume index, lipid accumulation product (LAP), and visceral adiposity index (VAI).

**Results:**

The analysis showed significant associations between all of these indices with incidence of DM (all *p* < 0.001). In the male participants, the strongest predictors for incidence of DM were LAP (AUC = 0.692), WHtR (AUC = 0.684), and WHR (AUC = 0.683). In the female participants, the strongest predictors were LAP (AUC = 0.744), WHtR (AUC = 0.710) and VAI (AUC = 0.710), followed by BRI (AUC = 0.708).

**Conclusion:**

Strong associations were found between the studied obesity-related indices and incidence of DM, and sex differences were found. Hence, to better control DM, reducing body weight may be beneficial in addition to lifestyle modifications, diet control, and pharmacological interventions.

## Introduction

Diabetes is one of the most prevalent metabolic disorders worldwide, and it is associated with severe complications and heavy financial and medical burdens. According to the 10th International Diabetes Federation Diabetes Atlas, the estimated global prevalence of diabetes among individuals aged 20 to 79 years in 2021 was 10.5% (536.6 million people) (1). According to the Taiwan Health Promotion Administration, the prevalence of diabetes mellitus (DM) in Taiwan between 2017 and 2020 was 11.05%, which is higher than the global prevalence (10.5%) (2).

Type 2 DM is the most common type of diabetes, characterized by insulin resistance, decrease in the number of beta cells, and hyperglycemia (3). Type 2 DM complications include cardiovascular diseases and other microvascular diseases affecting the kidneys, retina, and neurological system, leading to poor clinical outcomes (4). DM-related complications may pose a considerable economic burden on society even while having negative effects on society for patients and their families. Moreover, according to the cause of death statistics of 2021 from Taiwan Ministry of Health and Welfare, diabetes is one of Taiwan's top ten causes of death, and the number of deaths is increasing year by year (5). Therefore, it is of great importance to identify the risk factors associated with the development type 2 DM.

Many anthropometric indicators are simple and convenient tools for evaluating central obesity and the risk of metabolic syndrome. Such indicators include waist-to-hip ratio (WHR), waist-to-height ratio (WHtR), lipid accumulation product (LAP), body roundness index (BRI), visceral adiposity index (VAI), abdominal volume index (AVI), conicity index (CI), and body adiposity index (BAI) (6, 7). All of these anthropometric indicators can be calculated using simple clinical measurements such as waist circumference (WC), hip circumference (HC), body mass index (BMI), body height (BH), body weight (BW), triglycerides (TG), and high-density lipoprotein cholesterol (HDL-C). These obesity-related indices can evaluate obesity, which is defined by an excess accumulation of adipose tissue. Previous research has suggested the relationship between obesity with insulin resistance and type 2 diabetes, which is that non-esterified fatty acids secreted from adipose tissue in obese people may lead to insulin resistance and β-cell dysfunction (8). Our recent research revealed that these obesity indices are associated with fatty liver (9), albuminuria and advanced kidney disease (10), lung function (11), osteoporosis (12), hypertension (13), peripheral artery disease (14), and dementia (15). Previous studies have also demonstrated a relationship between obesity-related indices and DM (16–20). Nevertheless, few studies have investigated sex differences in the relationships between obesity-related indices and incidence of DM.

In this population-based cohort study, we enrolled over 26,000 participants from the Taiwan Biobank (TWB) and examined sex differences in the associations between obesity-related indices and incidence of DM. In addition, we determined the cutoff value of each obesity index to predict incidence of DM in men and women.

## Materials and methods

### Taiwan biobank

The population in Taiwan is rapidly aging, and hence the Ministry of Health and Welfare created the TWB to promote health care and prevent chronic diseases. The participants in the TWB are aged from 30 to 70 years and none have a previous diagnosis of cancer. Data available in the TWB include medical, genomic and lifestyle factors (21, 22). Ethical approval for the TWB was given by the Ethics and Governance Council of the TWB and Institutional Review Board on Biomedical Science Research, Academia Sinica, Taiwan.

During enrollment into the TWB, all participants provided data about their age and personal medical history (i.e. hypertension and DM). They also underwent physical examinations to obtain information on WC, HC, BW, BH and BMI. Fasting serum samples were obtained from all of the patients, and laboratory tests were conducted using an auto-analyzer (Roche Diagnostics GmbH, D-68298 Mannheim COBAS Integra 400). Laboratory data were also recorded at baseline after an 8-hour fast including: fasting glucose, glycated hemoglobin A1c (HbA1c), hemoglobin, TGs, total cholesterol, HDL-C, low-density lipoprotein cholesterol (LDL-C), estimated glomerular filtration rate (eGFR) [calculated using the 4-variable Modification of Diet in Renal Disease study equation (23)], and uric acid. Serum levels of creatinine were calculated using the compensated Jaffé (kinetic alkaline picrate) method using a calibrator that could traced in isotope-dilution mass spectrometry (24).

Systolic blood pressure (BP) and diastolic BP measurements were also performed in each participant with an automated BP monitor by a trained staff member. All measurements were made in triplicate after abstaining from smoking, caffeine, and exercise for at least 30 min. We used average BP measurements for analysis. Regular exercise was defined as exercising at least three times a week for at least 30 min each time, which is based on the projected “Physical Fitness 333 Plan,” promoted by the Ministry of Education in Taiwan in 1999 (25). Due to the widespread promotion, people in Taiwan still follow the “Physical Fitness 333 Plan” as a guideline for regular exercise. This study was conducted according to the Declaration of Helsinki, and approved by the Institutional Review Board of Kaohsiung Medical University Hospital [KMUHIRB-E(I)-20210058].

### Study design

This study is an observational cohort study.

### Sample population and sample size

**Figure 1:**
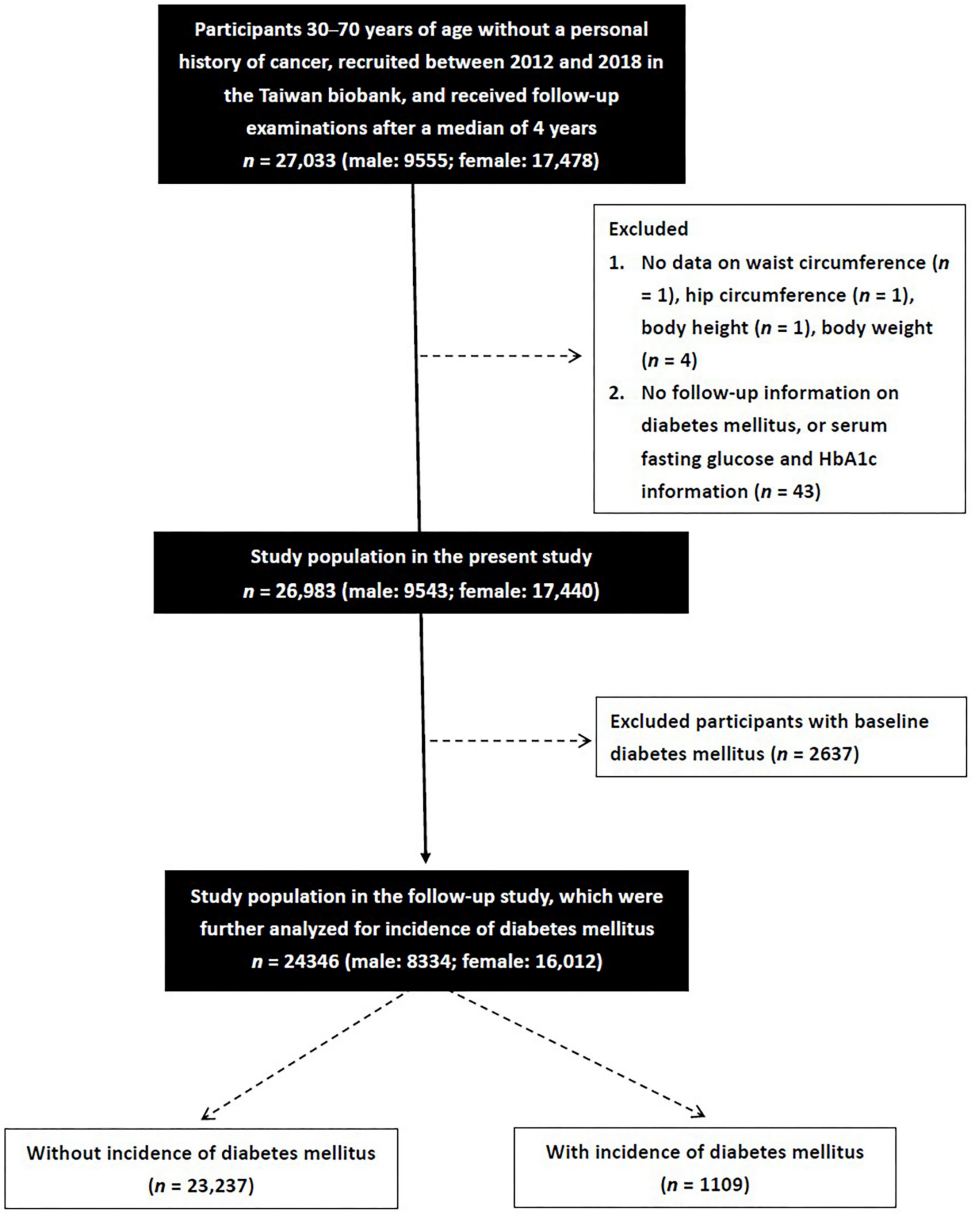
A total of 27,033 participants were screened. After exclusion those without waist circumference, hip circumference, body height, body weight, follow-up data on diabetes mellitus, serum fasting glucose or HbA1c, and those with baseline diabetes mellitus, the remaining 24,346 participants were enrolled.

A total of 27,033 participants (males: 9,555; females: 17,478) were screened, of whom had follow-up data for a median of 4 years and signed informed consent forms. Those with no data on WC (*n* = 1), HC (*n* = 1), BH (*n* = 1), and BW (*n* = 4), those with no follow-up data on DM, serum fasting glucose or HbA1c (*n* = 43), and those with baseline DM (*n* = 2,637) were excluded. The remaining 24,346 participants (males: 8,334; females: 16,012) were enrolled (Figure 1).

### Definition of incidence of DM

Participants with an HbA1c level <6.5%, fasting glucose level <126 mg/dL, and no self-reported past history of DM were classified into the non-DM group. The participants in whom DM developed during follow-up, defined as an HbA1c level ≥6.5%, fasting glucose level ≥126 mg/dL (26), or self-reported DM, were classified into the incidence of DM group.

### Calculation of obesity-related indices

1. BMI was calculated as:

$$\begin{array}{l} \text{BMI}=\text{BW}\left(\text{kg}\right)/\text{B}{\text{H}}^{2}\left(m\right) \end{array}$$

2. WHR was calculated as:

$$\begin{array}{l} \text{WHR}=\text{WC}\left(\text{cm}\right)/\text{HC}\left(\text{cm}\right) \end{array}$$

3. WHtR was calculated as:

$$\begin{array}{l} \text{WHtR}=\text{WC}\left(\text{cm}\right)/\text{BH}\left(\text{cm}\right) \end{array}$$

4. BRI was calculated as:

$$\begin{array}{l} \text{BRI }=364.2-365.5\;\times \sqrt{1-{\left(\frac{\frac{{\text{WC}}_{\left(\text{m}\right)}}{2\pi }}{0.5\;\times \;{\text{BH}}_{\left(\text{m}\right)}}\right)}^{2}} \end{array}$$

(27).

5. CI was calculated using the Valdez equation based on BW, BH and WC as:

$$\begin{array}{l} \text{CI}=\frac{{\text{WC}}_{\left(\text{m}\right)}}{0.109\;\times \sqrt{\frac{{\text{BW}}_{\left(\text{kg}\right)}}{{\text{BH}}_{\left(\text{m}\right)}}}} \end{array}$$

(28).

6. BAI was calculated according to the method of Bergman and colleagues as:

$$\begin{array}{l} \text{BAI }=\frac{{\text{HC}}_{\left(\text{cm}\right)}}{{{\text{BH}}_{\left(\text{m}\right)}}^{3/2}}\;-18\; \end{array}$$

(29).

7. AVI was calculated as

$$\begin{array}{l} \text{AVI}=\frac{2\;\times {\left(\;{\text{WC}}_{\left(\text{cm}\right)}\;\right)}^{2}+0.7\;\times {\left(\;{\text{WC}}_{\left(\text{cm}\right)}-\text{HC}{\;}_{\left(\text{cm}\right)}\right)}^{2}}{1000}\; \end{array}$$

(30).

8. LAP was calculated as:

$$\begin{array}{l} \text{LAP }=\left({\text{WC}}_{\left(\text{cm}\right)}\text{ − 65}\right)\times \;{\text{TG}}_{\left(\frac{\text{mmol}}{\text{L}}\right)}\text{inmalesandLAP}\\ \;=\left({\text{WC}}_{\left(\text{cm}\right)}\text{ − 58}\right)\times \;{\text{TG}}_{\left(\frac{\text{mmol}}{\text{L}}\right)}\text{infemales} \end{array}$$

(31).

9. VAI score was calculated as described previously (32) using the following sex-specific equations (with TG levels in mmol/l and HDL-cholesterol levels in mmol/l):

$$\begin{array}{l} \text{VAI }=\;\left(\frac{{\text{WC}}_{\left(\text{cm}\right)}}{\text{39}.\text{68 + }\left(\text{1}.\text{88 }\times \text{ BMI}\right)}\right)\times \left(\frac{{\text{TG}}_{\left(\frac{\text{mmol}}{\text{L}}\right)}}{\text{1}.\text{03}}\right)\\ \;\times \left(\frac{\text{1}.\text{31}}{{\text{HDL}}_{\left(\frac{\text{mmol}}{\text{L}}\right)}}\right)\text{ inmalesand}\\ \text{VAI }=\;\left(\frac{{\text{WC}}_{\left(\text{cm}\right)}}{\text{36}.\text{58 + }\left(\text{1}.\text{89 }\times \text{ BMI}\right)}\right)\times \left(\frac{{\text{TG}}_{\left(\frac{\text{mmol}}{\text{L}}\right)}}{\text{0}.\text{81}}\right)\\ \;\times \left(\frac{\text{1}.\text{52}}{{\text{HDL}}_{\left(\frac{\text{mmol}}{\text{L}}\right)}}\right)\text{ in females}. \end{array}$$

### Statistical analysis

SPSS (version 19, IBM Inc., Armonk, NY) was used for all statistical analyses. All continuous variables are expressed as mean ± SD, categorical variables as percentages, or medians (25^th^-75^th^ percentiles) were used to describe LAP and VAI. Normality tests were done to analyze the distribution of data collected for each group using the Kolmogorov-Smirnov. Homogeneity of variance was tested with the Levene test (Levene's test was used to assess the equality of variances, and an independent sample *t-*test). With respect to the comparison of data from different groups, the independent *t-*test was used for normally distributed variables, while the Mann–Whitney *U* test was used for non-normally distributed variables. Differences in categorical variables were examined by the chi-square test. Multivariable logistic regression analysis was performed to analyze associations between incidence of DM and the obesity-related indices, including all of the significant variables in univariate analysis. The natural logarithm was used for LAP and VAI. Receiver operating characteristic (ROC) curve analysis and areas under the ROC curves (AUCs) were used to assess the performance and predictive ability of the obesity-related indices for incidence of DM, respectively. The Optimal Cutoff Value was found as the cutoff with highest Youden Index, or equivalently, the highest Sensitivity + Specificity. *P* values < 0.05 were considered to be statistically significant.

## Results

The mean age of the 24,346 enrolled patients was 50.5 ± 10.4 years. The prevalence rate of incidence of DM was 5.7% in the males (*n* = 8,334) and 4.0% in the females (*n* = 16,012) (*p* < 0.001).

### Comparisons of the clinical characteristics between the male and female participants

Compared to the male participants, the female participants had lower prevalence rates of hypertension, smoking and alcohol history, lower values of systolic and diastolic BP, BH, BW, WC, HC, fasting glucose, HbA1c, hemoglobin, TGs, LDL-C and uric acid, and higher total cholesterol, HDL-C and eGFR (Table 1). The values of eight of the nine studied obesity-related indices (BMI, WHR, WHtR, BRI, CI, AVI, LAP, and VAI but not BAI) were lower in the female participants than in the male participants.

**Table 1:** Clinical characteristics of the study participants classified by sex.

**Characteristics**	**Male** **(*n* = 8,334)**	**Female** **(*n =* 16,012)**	***p***
Age (year)	50.6 ± 11.0	50.5 ± 10.1	0.376
Hypertension (%)	15.1	8.8	< 0.001
Smoking history (%)	58.2	7.5	< 0.001
Alcohol history (%)	7.0	0.7	< 0.001
Regular exercise habits (%)	48.3	47.4	0.188
Menstruation in female (%)	-	47.1	
Systolic BP (mmHg)	121.4 ± 16.1	113.9 ± 17.4	< 0.001
Diastolic BP (mmHg)	76.9 ± 10.4	69.7 ± 10.2	< 0.001
Body height (cm)	168.9 ± 6.2	157.1 ± 5.6	< 0.001
Body weight (Kg)	71.1 ± 10.7	57.6 ± 9.0	< 0.001
Waist circumference (cm)	86.7 ± 8.6	80.2 ± 9.2	< 0.001
Hip circumference (cm)	96.9 ± 6.3	94.8 ± 6.7	< 0.001
**Laboratory parameters**			
Fasting glucose (mg/dL)	94.4 ± 7.6	90.9 ± 7.4	< 0.001
HbA1c (%)	5.59 ± 0.34	5.56 ± 0.34	< 0.001
Hemoglobin (g/dL)	15.0 ± 1.1	13.0 ± 1.3	< 0.001
Triglyceride (mg/dL)	130.4 ± 97.8	98.7 ± 61.3	< 0.001
Total cholesterol (mg/dL)	192.7 ± 34.0	197.5 ± 35.3	< 0.001
HDL-C (mg/dL)	48.5 ± 11.0	58.3 ± 13.0	< 0.001
LDL-C (mg/dL)	123.2 ± 31.0	121.4 ± 31.4	< 0.001
eGFR (mL/min/1.73 m^2^)	99.1 ± 19.8	114.82 ± 25.6	< 0.001
Uric acid (mg/dL)	6.5 ± 1.4	4.9 ± 1.1	< 0.001
**Obesity-related indices**			
BMI (kg/m^2^)	24.9 ± 3.3	23.3 ± 3.4	< 0.001
WHR (%)	89.4 ± 5.4	84.5 ± 6.6	< 0.001
WHtR (%)	51.4 ± 5.1	51.1 ± 6.1	< 0.001
BRI	7.0 ± 1.6	6.4 ± 1.8	< 0.001
CI	1.23 ± 0.06	1.22 ± 0.09	< 0.001
BAI	26.2 ± 3.0	30.2 ± 3.8	< 0.001
AVI	15.3 ± 3.0	13.2 ± 3.0	< 0.001
LAP	34.4 ± 33.5	26.5 ± 23.3	< 0.001
VAI	1.7 ± 1.7	1.6 ± 1.4	< 0.001

DM, diabetes mellitus; BP, blood pressure; HbA1c, glycosylated hemoglobin A1c; HDL-C, high-density lipoprotein cholesterol; LDL-C, low-density lipoprotein cholesterol; eGFR, estimated glomerular filtration rate; BMI, body mass index; WHR, waist–hip ratio; WHtR, waist-to-height ratio; BRI, body roundness index; CI, conicity index; BAI, body adiposity index; AVI, abdominal volume index; LAP, lipid accumulation product; VAI, visceral adiposity index.

### Comparisons of the clinical characteristics between the male and female participants with and without incidence of DM

The male participants with incidence of DM were older and had higher rates of hypertension and a history of smoking and alcohol consumption than those without incidence of DM (Table 2). In addition, the male participants with incidence of hypertension had higher systolic and diastolic BPs, BH, BW, WC, HC, fasting glucose, HbA1c, TGs, total cholesterol, LDL-C and uric acid, and lower HDL-C and eGFR than those without incidence of DM. Moreover, the male participants with incidence of DM had higher values of all nine obesity-related indices than those without incidence of DM.

**Table 2:** Clinical characteristics of the study participants classified by the presence of different sex and incidence of DM.

**Characteristics**	**Male (*n* = 8,334)**			**Female (*n* = 16,012)**		
	**Non-diabetic** **(*****n*** = **7,858)**	**Diabetic** **(*****n** =* **476)**	***p***	**Non-diabetic** **(*****n** =* **15,379)**	**Diabetic** **(*****n** =* **633)**	***p***
Age (year)	50.4 ± 11.0	53.9 ± 9.9	< 0.001	50.3 ± 10.1	55.3 ± 8.3	< 0.001
Hypertension (%)	14.3	29.0	< 0.001	8.2	22.9	< 0.001
Smoking history (%)	57.9	64.3	0.006	7.5	6.2	0.201
Alcohol history (%)	6.8	10.5	0.002	0.7	0.3	0.446
Regular exercise habits (%)	48.4	45.6	0.228	47.2	51.0	0.061
Menstruation in female (%)	-	-	-	47.9	27.8	< 0.001
Systolic BP (mmHg)	121.1 ± 16.0	126.4 ± 16.6	< 0.001	113.5 ± 17.2	124.4 ± 18.5	< 0.001
Diastolic BP (mmHg)	76.8 ± 10.4	79.2 ± 10.3	< 0.001	69.6 ± 10.2	74.1 ± 10.3	< 0.001
Body height (cm)	169.0 ± 6.3	167.6 ± 6.1	< 0.001	157.1 ± 5.6	155.9 ± 5.2	< 0.001
Body weight (Kg)	70.8 ± 10.5	75.2 ± 12.4	< 0.001	57.4 ± 8.9	62.4 ± 9.7	< 0.001
Waist circumference (cm)	86.4 ± 8.5	91.6 ± 9.0	< 0.001	80.0 ± 9.1	86.5 ± 9.2	< 0.001
Hip circumference (cm)	96.8 ± 6.2	98.7 ± 7.2	< 0.001	94.7 ± 6.7	97.2 ± 7.2	< 0.001
**Laboratory parameters**						
Fasting glucose (mg/dL)	93.9 ± 7.2	102.8 ± 9.8	< 0.001	90.5 ± 7.0	100.5 ± 10.0	< 0.001
HbA1c (%)	5.57 ± 0.33	6.01 ± 0.29	< 0.001	5.54 ± 0.33	6.04 ± 0.28	< 0.001
Hemoglobin (g/dL)	15.0 ± 1.1	15.1 ± 1.3	0.118	13.0 ± 1.3	13.3 ± 1.2	< 0.001
Triglyceride (mg/dL)	127.3 ± 90.8	181.9 ± 168.5	< 0.001	96.9 ± 58.6	142.3 ± 95.4	< 0.001
Total cholesterol (mg/dL)	192.4 ± 33.8	197.6 ± 38.1	0.001	197.1 ± 35.2	207.3 ± 36.0	< 0.001
HDL-C (mg/dL)	48.8 ± 11.1	43.8 ± 8.9	< 0.001	58.5 ± 13.0	52.4 ± 11.6	< 0.001
LDL-C (mg/dL)	123.0 ± 30.8	126.3 ± 34.2	0.023	121.0 ± 31.3	130.9 ± 33.1	< 0.001
eGFR (mL/min/1.73 m^2^)	99.2 ± 19.8	96.9 ± 20.6	0.018	114.9 ± 25.7	112.9 ± 24.1	0.041
Uric acid (mg/dL)	6.5 ± 1.3	6.9 ± 1.5	< 0.001	4.9 ± 1.1	5.5 ± 1.1	< 0.001
**Obesity-related indices**						
BMI (kg/m^2^)	24.8 ± 3.1	26.7 ± 3.6	< 0.001	23.2 ± 3.4	25.7 ± 3.7	< 0.001
WHR	0.89 ± 0.05	0.93 ± 0.05	< 0.001	0.84 ± 0.07	0.89 ± 0.06	< 0.001
WHtR	0.51 ± 0.05	0.55 ± 0.05	< 0.001	0.51 ± 0.06	0.56 ± 0.06	< 0.001
BRI	7.0 ± 1.6	8.0 ± 1.8	< 0.001	6.3 ± 1.8	7.7 ± 2.0	< 0.001
CI	1.23 ± 0.06	1.26 ± 0.06	< 0.001	1.22 ± 0.08	1.26 ± 0.08	< 0.001
BAI	26.1 ± 3.0	27.5 ± 3.2	< 0.001	30.2 ± 3.7	32.0 ± 3.9	< 0.001
AVI	15.2 ± 2.9	17.0 ± 3.4	< 0.001	13.1 ± 2.9	15.2 ± 3.3	< 0.001
LAP	33.0 ± 31.4	56.4 ± 53.2	< 0.001	25.7 ± 22.4	46.3 ± 34.0	< 0.001
VAI	1.6 ± 1.5	2.6 ± 3.2	< 0.001	1.5 ± 1.3	2.6 ± 2.4	< 0.001

DM, diabetes mellitus; BP, blood pressure; HbA1c, glycosylated hemoglobin A1c; HDL-C, high-density lipoprotein cholesterol; LDL-C, low-density lipoprotein cholesterol; eGFR, estimated glomerular filtration rate; BMI, body mass index; WHR, waist–hip ratio; WHtR, waist-to-height ratio; BRI, body roundness index; CI, conicity index; BAI, body adiposity index; AVI, abdominal volume index; LAP, lipid accumulation product; VAI, visceral adiposity index.

The female participants with incidence of DM were older, had a higher prevalence rate of hypertension, lower menstruation status, higher systolic and diastolic BPs, BW, WC, HC, fasting glucose, HbA1c, hemoglobin, TGs, total cholesterol, LDL-C and uric acid, and lower BH, HDL-C and eGFR than those without incidence of DM. Moreover, the female participants with incidence of DM had higher values of all studied obesity-related indices than those without incidence of DM.

### Associations among obesity-related indices with incidence of DM by sex

The following multivariable logistic regression models were used to examine the associations between each obesity-related index with incidence of DM by sex:

1. For WHtR, WHR, CI, BRI, BMI, BAI, and AVI: adjustments for age, hypertension, smoking and alcohol history, systolic and diastolic BPs, hemoglobin, TGs, total cholesterol, HDL-C, LDL-C, eGFR and uric acid in males (significant variables in Table 2); age, hypertension, menstruation status, systolic and diastolic BPs, hemoglobin, TGs, total cholesterol, HDL-C, LDL-C, eGFR and uric acid in females (significant variables in Table 2).
2. For LAP: the same adjustments as in model 1 except for TGs.
3. For VAI: the same adjustments as in model 1 except for TGs and HDL-C.

The results showed that high BMI (per 1 kg/m^2^; odds ratio [OR] = 1.140), WHR (per 0.01; OR = 1.082), WHtR (per 0.01; OR = 1.088), BRI (per 1; OR = 1.263), CI (per 0.1; OR = 1.425), BAI (per 1; OR = 1.082), AVI (per 1; OR = 1.124), LAP (log per 1; OR = 8.951) and VAI (log per 1; OR = 9.104) were significantly associated with incidence of DM in the male participants (all *p* < 0.001) (Table 3). Similarly, high BMI (per 1 kg/m^2^; OR = 1.099), WHR (per 0.01; OR = 1.051), WHtR (per 0.01; OR = 1.056), BRI (per 1; OR = 1.171), CI (per 0.1; OR = 1.233), BAI (per 1; OR = 1.043), AVI (per 1; OR = 1.095), LAP (log per 1; OR = 8.687), and VAI (log per 1; OR = 6.629) were significantly associated with incidence of DM in the female participants (all *p* < 0.001).

**Table 3:** Association of obesity-related indices with incidence of DM using multivariable logistic regression analysis.

**Obesity-related indices**	**Male (*n* = 8,334)**			**Female (*n* = 16,012)**		
	**Multivariable**			**Multivariable**		
	**OR**	**95% confidence interval**	***p***	**OR**	**95% confidence interval**	***p***
BMI (per 1 kg/m^2^)^a^	1.140	1.105–1.175	< 0.001	1.099	1.073–1.125	< 0.001
WHR (per 0.01)^a^	1.082	1.061–1.104	< 0.001	1.051	1.037–1.065	< 0.001
WHtR (per 0.01)^a^	1.088	1.066–1.110	< 0.001	1.056	1.041–1.070	< 0.001
BRI (per 1)^a^	1.263	1.193–1.337	< 0.001	1.171	1.123–1.221	< 0.001
CI (per 0.1)^a^	1.425	1.208–1.682	< 0.001	1.233	1.118–1.359	< 0.001
BAI (per 1)^a^	1.082	1.048–1.117	< 0.001	1.043	1.022–1.066	< 0.001
AVI (per 1)^a^	1.124	1.089–1.159	< 0.001	1.095	1.068–1.123	< 0.001
LAP (log per 1)^b^	8.951	5.454–14.689	< 0.001	8.687	5.782–13.052	< 0.001
VAI (log per 1)^c^	9.104	6.141–13.497	< 0.001	6.629	4.864–9.035	< 0.001

Values expressed as odds ratio (OR) and 95% confidence interval. DM, diabetes mellitus; BMI, body mass index; WHR, waist–hip ratio; WHtR, waist-to-height ratio; BRI, body roundness index; CI, conicity index; BAI, body adiposity index; AVI, abdominal volume index; LAP, lipid accumulation product; VAI, visceral adiposity index.

a Covariates in the multivariable model included age, hypertension, smoking and alcohol history, systolic and diastolic BPs, hemoglobin, triglyceride, total cholesterol, HDL-C, LDL-C, eGFR and uric acid in male (significant variables in Table 2); age, hypertension, menstruation status, systolic and diastolic BPs, hemoglobin, triglyceride, total cholesterol, HDL-C, LDL-C, eGFR and uric acid in female (significant variables in Table 2).

b Covariates as ^a^Covariates, except for triglyceride.

c Covariates as ^a^Covariates, except for triglyceride and HDL-C.

Significant interactions were found between sex and WHR (β = −0.024; *p* = 0.022), CI (β = −0.232; *p* = 0.008), LAP (β = 0.008; *p* < 0.001), and VAI (β = 0.092; *p* = 0.001) on incident DM. However, no significant differences were found in the other indices. In the men, WHR and CI were more strongly correlated with incidence of DM than in the women, whereas LAP and VAI were more closely linked with incidence of DM in the women than in the men.

### Performance and predictive ability of the obesity-related indices to identify incidence of DM in the male and female participants

**Figure 2:**
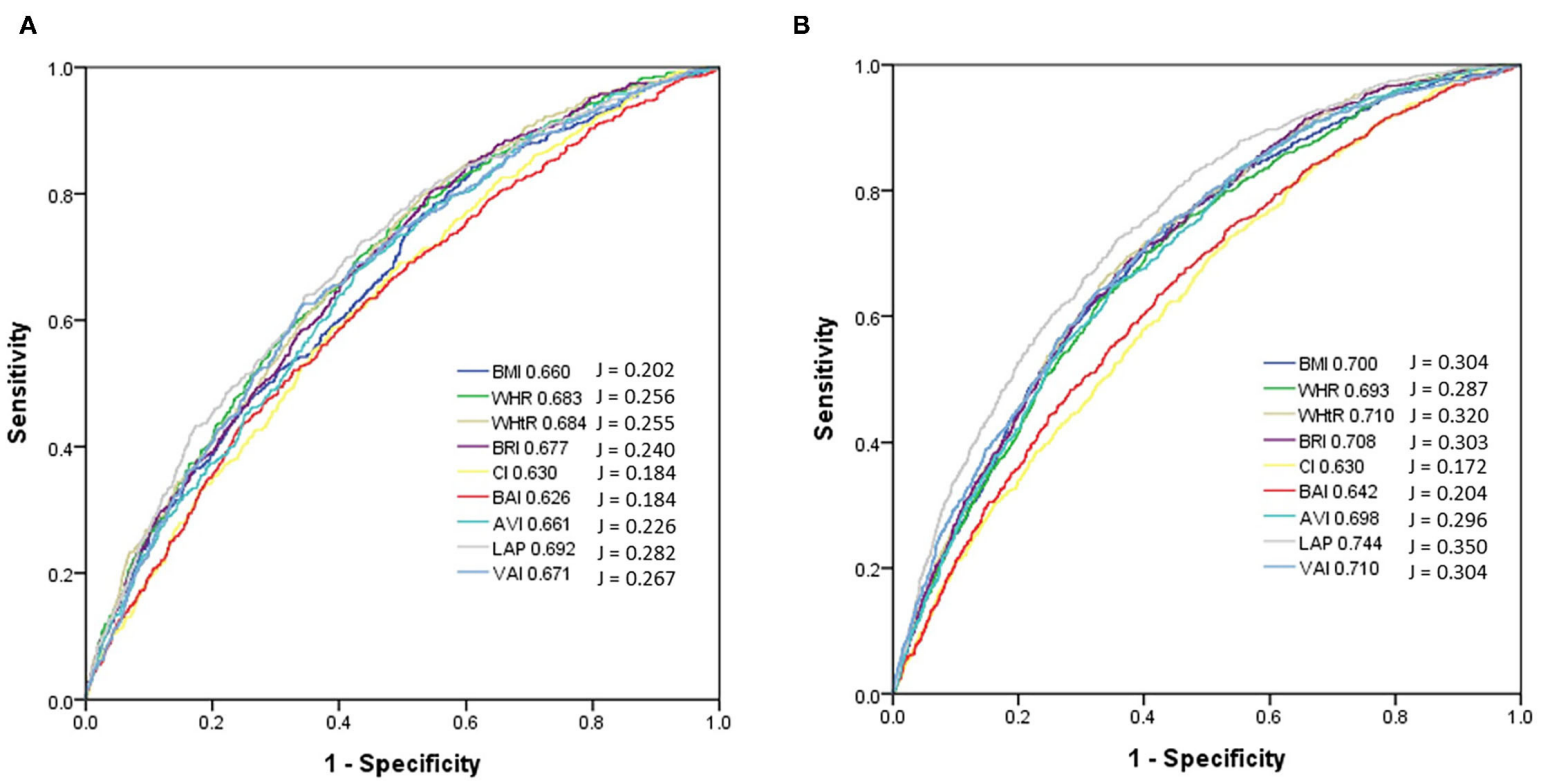
Comparison of the AUC and J values of 9 obesity-related indices for diagnosis of incidence of diabetes mellitus among **(A)** males and **(B)** females. In the male participants, LAP had the highest AUC (0.692) and J (0.282), followed by WHtR (AUC = 0.684, J = 0.255), WHR (AUC = 0.683, J = 0.256), BRI (AUC = 0.677, J = 0.240), VAI (AUC = 0.671, J = 0.267), AVI (AUC = 0.661, J = 0.226), BMI (AUC = 0.660, J = 0.202), CI (AUC = 0.630, J = 0.184), and BAI (AUC = 0.626, J = 0.184). In the female participants, LAP also had the highest AUC (0.744) and J (0.350), followed by WHtR (AUC = 0.710, J = 0.320), VAI (AUC = 0.710, J = 0.304), BRI (AUC = 0.708, J = 0.303), BMI (AUC = 0.700, J = 0.304), AVI (AUC = 0.698, J = 0.296), WHR (AUC = 0.693, J = 0.287), BAI (AUC = 0.642, J = 0.204), and CI (AUC = 0.630, J = 0.172).

The performance (ROC curves), predictive ability (AUCs) and J value of the obesity-related indices to identify incidence of DM in the male and female participants were analyzed (Figure 2). Table 4 showed that in the male participants, LAP had the highest AUC (0.692), followed by WHtR (0.684), WHR (0.683), BRI (0.677), VAI (0.671), AVI (0.661), BMI (0.660), CI (0.630), and BAI (0.626). In addition, Table 5 showed that in the female participants, LAP also had the highest AUC (0.744), followed by WHtR (0.710), VAI (0.710), BRI (0.708), BMI (0.700), AVI (0.698), WHR (0.693), BAI (0.642), and CI (0.630).

**Table 4:** Area under curve (AUC), cutoff value, sensitivity, specificity, and Youden index of 9 obesity-related indices for incidence of DM in male participants.

**Obesity-related indices**	**AUC** **(95% confidence interval)**	**Cutoff** **value**	**Sensitivity (%)**	**Specificity (%)**	**Youden index**
BMI (kg/m^2^)	0.660 (0.635–0.685)^*^	25.287	60.1	60.0	0.202
WHR	0.683 (0.659–0.707)^*^	0.908	62.8	62.8	0.256
WHtR	0.684 (0.661–0.708)^*^	0.526	62.8	62.7	0.255
BRI	0.677 (0.653–0.701)^*^	7.312	62.0	62.0	0.240
CI	0.630 (0.605–0.655)^*^	1.242	59.2	59.2	0.184
BAI	0.626 (0.600–0.652)^*^	26.676	59.2	59.2	0.184
AVI	0.661 (0.637–0.685)^*^	15.712	61.3	61.3	0.226
LAP	0.692 (0.668–0.716) ^*^	32.649	64.1	64.1	0.282
VAI	0.671 (0.647–0.696)^*^	1.549	63.4	63.3	0.267

^*^p < 0.001. DM, diabetes mellitus; BMI, body mass index; WHR, waist–hip ratio; WHtR, waist-to-height ratio; BRI, body roundness index; CI, conicity index; BAI, body adiposity index; AVI, abdominal volume index; LAP, lipid accumulation product; VAI, visceral adiposity index.

**Table 5:** Area under curve (AUC), cutoff value, sensitivity, specificity, and Youden index of 9 obesity-related indices for incidence of DM in female participants.

**Obesity-related indices**	**AUC** **(95% confidence interval)**	**Cutoff** **value**	**Sensitivity (%)**	**Specificity (%)**	**Youden index**
BMI (kg/m^2^)	0.700 (0.680–0.720)^*^	24.008	65.2	65.2	0.304
WHR	0.693 (0.673–0.713)^*^	0.864	64.3	64.4	0.287
WHtR	0.710 (0.691–0.729)^*^	0.529	66.0	66.0	0.320
BRI	0.708 (0.689–0.727)^*^	6.753	65.2	65.1	0.303
CI	0.630 (0.609–0.651)^*^	1.229	58.6	58.6	0.172
BAI	0.642 (0.620–0.663)^*^	30.689	60.2	60.2	0.204
AVI	0.698 (0.679–0.717)^*^	13.792	64.8	64.8	0.296
LAP	0.744 (0.725–0.762)^*^	27.819	67.5	67.5	0.350
VAI	0.710 (0.690–0.729)^*^	1.495	65.2	65.2	0.304

^*^p < 0.001. Abbreviations are the same as in Table 1. DM, diabetes mellitus; BMI, body mass index; WHR, waist–hip ratio; WHtR, waist-to-height ratio; BRI, body roundness index; CI, conicity index; BAI, body adiposity index; AVI, abdominal volume index; LAP, lipid accumulation product; VAI, visceral adiposity index.

The AUC, cutoff, sensitivity, specificity, and Youden index values of the obesity-related indices to identify incidence of DM in the male and female participants are shown in Tables 4, 5, respectively. In male participants, LAP had the highest Youden index (0.282), followed by VAI (0.267) and WHR (0.256). In addition, in female participants, LAP had the highest Youden index (0.282), followed by WHtR (0.320), BMI (0.304), and VAI (0.256).

## Discussion

In this study, we investigated sex differences in the associations between nine obesity-related indices with incidence of DM after a median 4-year follow-up period. We found that all of the studied obesity indices were associated with incidence of DM in both the male and female participants. Furthermore, there were sex differences in the relationships between some of the obesity indices and incidence of DM. In the men, WHR and CI were more strongly correlated with incidence of DM than in the women, whereas LAP and VAI were more closely linked with incidence of DM in the women than in the men.

Our results demonstrate the predictive ability of obesity-related indices for incidence of DM. In the male participants, the strongest predictors for incidence of DM were LAP, WHtR, and WHR. In the female participants, the strongest predictors were LAP, WHtR, and VAI, followed by BRI. Previous epidemiologic studies have reported an association between central obesity and the incidence of DM (16–18, 33). Many obesity-related indices can be used to assess the degree of central obesity, of which LAP had the highest predictive ability for incidence of DM in both the male and female participants in the present study. In a Japanese study of 10,170 middle-aged individuals, a high LAP was associated with DM, with an OR of 19.09 (95% confidence interval: 6.57–55.50) in women and 7.40 (95% confidence interval: 5.10–10.75) in men (18). A 6-year cohort study conducted by Bozorgmanesh et al. with a total of 5,018 non-diabetic subjects also found that LAP was a strong predictor of incidence of DM in both sexes (34). LAP has two important components, namely WC and serum TG level, which reflect abdominal fat and lipid metabolism (35). Increased visceral fat and serum TG levels were linked with insulin resistance in a Japanese study of metabolically obese subjects with normal weight and glucose tolerance (36). The association between visceral fat and DM could be because adipocytokines are released by visceral fat rather than subcutaneous adipose tissue, and their circulating levels are associated with the development of insulin resistance (36, 37). Growing evidence supports that WHtR has a significant relationship with new-onset DM (38–41), and a meta-analysis found that WHtR had a higher predictive power for the risk of DM than WC and BMI (38). This could be because BMI only measures total BW and cannot discriminate between visceral adipose and skeletal muscle mass (40), while WC does not take tall and short body shapes into account. Other research has revealed an important and independent association between WHR and DM (17, 42, 43). However, the use of WHR is controversial due to its uncertain biologic interpretation, lack of sensitivity to weight gain, and high variability across age, sex, and different populations (17). Many studies have shown that BRI can be used to predict visceral fat (27), metabolic syndrome (44) and DM (19, 45). In a prospective cohort study, BRI and WHtR were shown to have a similar predictive ability for DM in hypertensive patients, possibly because both BRI and WHtR are calculated based on WC and height (46). Another useful predictor of DM is VAI, which was first described by Amato et al. as visceral adipose dysfunction associated with cardiometabolic risk in a healthy population (47). Previous studies have reported that VAI may predict DM (20, 48), and that it is an indicator of adipose tissue dysfunction (49). In addition, other studies have reported a positive correlation between VAI and adipocytokines, and this may explain the pathogenesis of developing insulin resistance (50, 51).

We also found sex differences in the predictive ability of the obesity-related indices for incidence of DM. Previous studies have demonstrated sex differences in fat distribution, and the type of fat deposition has been shown to have a varied effect on cardiometabolic risk (52–54). Many mechanisms, including fat microenvironment, cell-intrinsic features, and sex hormones have been postulated to explain the sex dimorphism in adipose distribution (52). In men, fat deposits are more likely to be centrally distributed or apple-shaped, which is correlated with an increased risk of cardiometabolic disease. In women, fat deposits are prone to peripheral fat distribution or pear-shaped, which may protect against cardiometabolic disease (52, 54). Prior studies have reported sex differences in lipid profiles. Compared to men, premenopausal women have higher levels of serum HDL, and lower levels of LDL, very-low-density lipoprotein and TGs, which may lead to a protective effect against cardiovascular disease by estrogen (55). On the other hand, excess visceral fat has been closely correlated with the impaired inhibition of free fatty acid release in response to insulin, along with hypertriglyceridemia and low HDL-C concentrations (56). Because women have a tendency to accumulate peripheral fat, elevated serum TGs along with reduced HDL-C, but not WC, may be better able to identify visceral adipocyte expansion in women. LAP is proportional to TG concentration and VAI is dependent on TG and HDL-C levels. Both can indicate the presence of extra lipid fuel and ectopic fat deposits, which increase the risk of metabolic disease. Our results suggest that abnormal lipid metabolism increases the risk of developing DM in women. In summary, this study suggested WHR and CI are more specific to men for predicting the development of type 2 DM, while LAP and VAI are more specific to women.

The strengths of this study include the large population-based investigation and follow-up of sex differences in the associations between nine obesity-related indices and incidence of DM. However, there were also some limitations. First, data on medications and other factors which could affect the findings of incidence of DM such as anti-diabetics, anti-hypertensives, lipid-lowering agents and proteinuria are not recorded in the TWB. This may have led to underestimation of the association between incidence of DM and the obesity-related indices. Second, this study was conducted in Taiwan with participants of Chinese ethnicity, and this may limit the generalizability of our findings. Third, as only approximately 25% of the enrollees in the TWB undergo follow-up evaluations, sample bias is possible.

In conclusion, we found that all nine studied obesity-related indices were significant predictors of incidence of DM, and we also found differences in the associations between the male and female participants. Hence, to better control DM, reducing body weight may be beneficial in addition to lifestyle modifications, diet control, and pharmacological interventions.

## Data availability statement

The raw data supporting the conclusions of this article will be made available by the authors, without undue reservation.

## Ethics statement

The studies involving human participants were reviewed and approved by Institutional Review Board of Kaohsiung Medical University Hospital [KMUHIRB-E(I)-20210058]. The patients/participants provided their written informed consent to participate in this study.

## Author contributions

Conceptualization, methodology, validation, formal analysis, writing—review and editing, supervision, software, investigation, resources, project administration, funding acquisition, and visualization: S-CC. Data curation: T-LC, Y-HL, P-YW, J-CH, and S-CC. Writing—original draft preparation: T-LC and S-CC. All authors have read and agreed to the published version of the manuscript.
